# P2X7 Receptor: an Emerging Target in Alzheimer’s Disease

**DOI:** 10.1007/s12035-023-03699-9

**Published:** 2023-11-09

**Authors:** Qiang Huang, Jun Ying, Wen Yu, Yao Dong, Hao Xiong, Yiping Zhang, Jie Liu, Xifeng Wang, Fuzhou Hua

**Affiliations:** 1 Department of Anesthesiology, the Second Affiliated Hospital of Nanchang University, 1# Minde Road, Nanchang, 330006 Jiangxi China; 2 Key Laboratory of Anesthesiology of Jiangxi Province, 1# Minde Road, 330006 Nanchang City, Jiangxi Province People’s Republic of China; 3 Department of Anesthesiology, the First Affiliated Hospital of Nanchang University, 17# Yongwai Road, Nanchang, 330006 Jiangxi China

**Keywords:** Alzheimer’s disease, P2X7R, Mitochondrial dysfunction, Neuroinflammation, Oxidative stress

## Abstract

Alzheimer’s disease (AD) is a major cause of age-related dementia, which is becoming a global health crisis. However, the pathogenesis and etiology of AD are still not fully understood. And there are no valid treatment methods or precise diagnostic tools for AD. There is increasing evidence that P2X7R expression is upregulated in AD and is involved in multiple related pathological processes such as Aβ plaques, neurogenic fiber tangles, oxidative stress, and chronic neuroinflammation. This suggests that P2X7R may be a key player in the development of AD. P2X7R is a member of the ligand-gated purinergic receptor (P2X) family. It has received attention in neuroscience due to its role in a wide range of aging and age-related neurological disorders. In this review, we summarize current information on the roles of P2X7R in AD and suggest potential pharmacological interventions to slow down AD progression.

## Introduction

Alzheimer’s disease (AD) is an age-related neurodegenerative disease characterized by overall cognitive decline, including progressive loss of memory, orientation, and reasoning skills. In line with increasing life expectancy, the 2020 census found that 18.67% of the total population of China (approximately 264 million) is over the age of 60 and that number will grow, projected to reach 500 million by 2050. The risk of developing AD increases exponentially with age, and society will bear an increasing burden due to AD unless effective prevention and treatment strategies are developed [1].

In the past decades, the Aβ hypothesis and the tau hypothesis were promulgated as the main explanations for the pathogenesis of Alzheimer’s disease [2]. However, the pathological features of AD are not only characterized by Aβ plaques, neurogenic fiber tangles (NFT). Studies now find that AD is always accompanied by synaptic loss and inflammation [3, 4]. Not only that, studies have shown that mitochondrial dysfunction and oxidative stress are involved in the pathogenesis of AD [5–7]. Even in the absence of Aβ plaques and tau tangles, mitochondrial dysfunction is one of the earliest prominent features of AD. Therefore, it is now generally accepted that AD is a multifactorial neurodegenerative disease involving different pathological processes. However, the pathogenesis and etiology of AD are still not fully understood. And there are no valid treatment methods or precise diagnostic tools for AD.

Therefore, there is an urgent need to find new treatment pathways to reduce the progression of the disease. The purinergic receptors have been of interest for a long time in neurodegenerative diseases [8, 9]. The purinergic receptor family can be divided into two main types, P2X and P2Y, respectively. P2X7R is a family member of P2X. It is widely expressed in the nervous system and is involved in a variety of neurological functions [10]. Not only that, P2X7R is involved in the progression of multiple diseases including Parkinson’s disease, multiple sclerosis, and Huntington’s disease [11–13]. These had intrigued researchers about the role of P2X7R in AD. Researchers find P2X7R involved in multiple processes in the progression of AD. Firstly, P2X7R can regulate Aβ formation and the researchers found that the use of P2X7R antagonists reduced plaques in J20 mice [14]. P2X7R is inextricably linked to tau protein phosphorylation, oxidative stress, or chronic neuroinflammatory pathological processes in AD [15–17]. These indicate that this P2X7R may become a drug target for a new AD therapeutic approach.

Here, we summarize the molecular characteristics, structure, and features of P2X7R. Subsequently, we discuss the significance of P2X7R in the pathogenesis of AD. Finally, a brief review of the research progress on P2X7R antagonists is presented.

## The Structure and Molecular Physiology of P2X7R

**Fig. 1:**
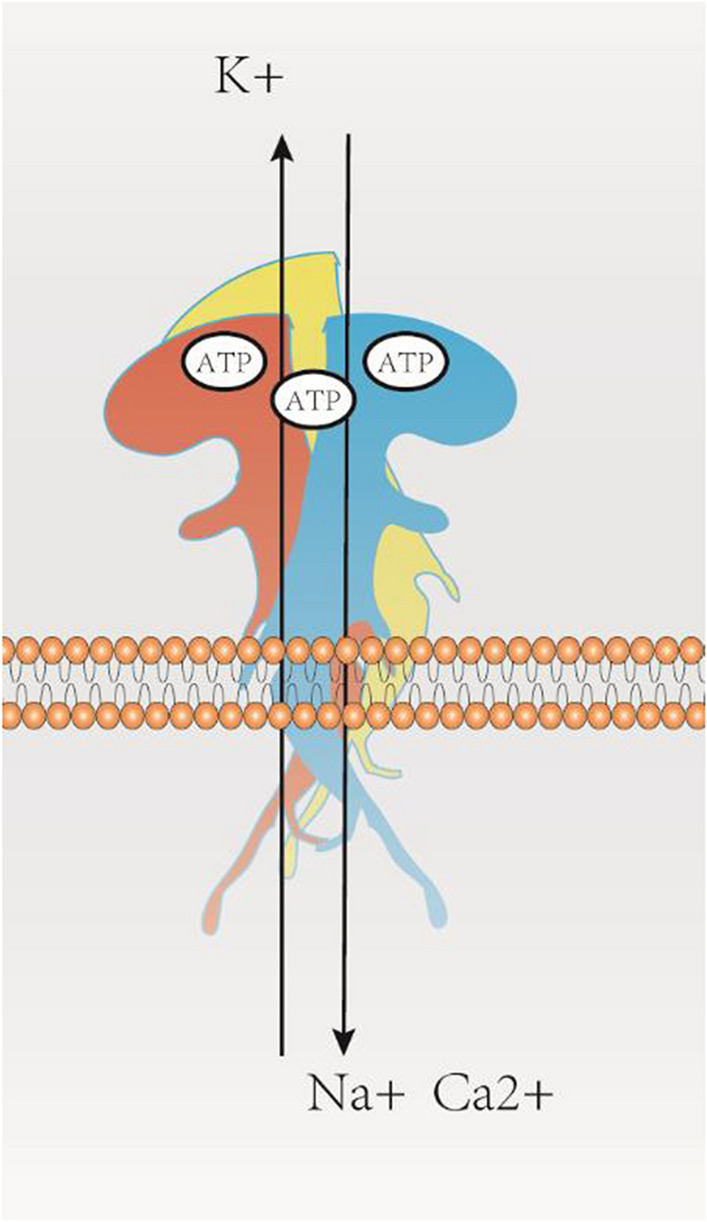
The P2X7R functions as a homo-trimer, forming a chalice-like structure, while the individual P2X7R subunit is akin to a leaping dolphin. P2X7R is a non-selective cation channel that is activated by high concentrations of ATP. Transient activation leads to the opening of the cation channel, allowing K^+^ efflux and Na^+^ influx into the cell, resulting in an inward current/depolarization at the resting membrane potential

Adenosine 5-triphosphate (ATP) is the main energy carrying molecule in cells. However, studies now show that ATP can also play an important role in the nervous system as a neurotransmitter. Purinergic receptors were first described in 1976, and as research progressed, researchers have so far identified multiple purinergic receptors, including seven P2X receptor subtypes and eight P2Y receptor subtypes [18]. Ionotropic P2X7R belongs to the P2X purinoceptor family [19]. Similar to other P2XR types, P2X7R is an ATP-gated, non-selective homotrimeric cation channel [20]. However, P2X7R also has several features that are significantly different from other members of the P2X receptor family and deserve special attention. P2X1-7 receptors are homo-trimeric forms formed from three identical P2X subunits. In addition, some P2X receptors exist as heterotrimeric [21]. P2X7R acts via the homotrimeric form. The common structural motifs of P2X7R are two transmembrane structural domains (TM1, TM2) [22]. It contains a large, glycosylated, cysteine-rich extracellular loop, and a short intracellular N-terminal domain. Moreover, an intracellular C-terminal structural domain is longer than in other P2X receptor subunits [23]. The molecular structure of a single P2X7R subunit resembles a jumping dolphin. When co-assembled as a homotrimer, P2X7R has a cup-like structure [24]. Notably, P2X7R is only activated by high concentrations of ATP in the millimolar range, which is significantly higher than that required to activate other purinoceptor channels [19]. In addition, it is a non-selective cation channel. It shows different reactions to the agonist based on its concentration and time of application. A brief activation, resulting in the opening of cation channels, allows K^+^ efflux as well as Ca^2+^ and Na^+^ influx into the cell, leading to an inward current/depolarization at the resting membrane potential** (**Fig. 1). By contrast, prolonged activation of P2X7R by agonists leads to the formation of a large aqueous pore, which is permeable to molecules with molecular masses up to 900 Da [25–28]. This ultimately leads to membrane blebbing, cytokine release, and cell death [29, 30]. However, the underlying molecular mechanism is still a hotly debated issue. There are two main possible mechanism hypotheses. The first hypothesis suggests an asymptotic expansion of P2X7R-gated channels, in which a second transmembrane structural domain in P2X7R is thought to be critical for pore formation [31]. Another piece of evidence in favor of this hypothesis is that negatively charged fluorescent dyes with molecular diameters up to 1.4 nm pass directly through the P2X7R channel [32]. Another hypothesis suggests an association with a pore-forming protein, pannexin-1 hemichannel. It was shown that in the absence of pannexin-1 hemichannel, the ability of astrocytes to take up macromolecular dyes was significantly reduced [33]. But some studies have found the opposite [34]. One possible explanation is that the P2X7R splice variant shows different pore-forming properties [35, 36].

## The Function of P2X7R in the CNS

### P2X7R in Microglia

P2X7R was early found to be expressed in immune cells. Notably, P2X7R mediates multiple physiological aspects of microglia. A recent study unexpectedly found that P2X7R activation promotes the migratory capacity and phagocytosis of microglia [37]. Another study showed that the key enzyme for microglia migration is glycogen synthase-3 [38]. Indeed, P2X7R antagonists were found to reduce the number of amyloid plaques in the rat hippocampus, which is also thought to be associated with reduced GSK3 activity in microglia [14]. The large number of microglia with high P2X7R expression clustered around senile plaques in postmortem brain samples of AD patients and in AD mouse models may also be closely related to this phenomenon [39, 40]. In addition, activation of P2X7R drives microglial activation and is a key factor in microglial proliferation [41]. Experiments in rat primary hippocampal neurons show that P2X7R drives microglial activation and can promote microglial proliferation [42]. More importantly, P2X7R is an important regulator of microglial secretion of pro-inflammatory cytokines and chemokines. For example, P2X7R is not only involved in the maturation of IL-1β in microglia but also plays an important role in its release [16, 43].

### P2X7R in Neurons

The localization of P2X7R on neurons has been the subject of a long-standing debate, and even now there is no universal consensus. At first, researchers found that P2X7R immunoreactivity was significantly expressed on excitatory nerve terminals [44]. Yu et al. used isotope in situ hybridization to examine the distribution of P2X7R mRNAs in the brain and found that P2X7 mRNA signals were also detected on NeuN-positive neurons cells [45]. During brain growth, both neural precursor cells and neuroblastoma cells can express P2X7R. Many studies have reported the presence of functional P2X7R, including embryonic stem cells, neuron-like human embryonic stem cells-derived neural progenitor cells, human neural progenitor cells, as well as NPCs isolated from the subventricular zone of adult mice or the striatum of embryonic mice [46–49]. However, immunohistochemical studies showed that P2X7R was expressed at the cell membrane of microglia and NPC but not on neurons [50]. In contrast to the contradictory results of immunohistochemistry, the function of the P2X7 receptor in the nervous system was demonstrated. P2X7R is involved in the growth of neuronal axons [51], and further experiments demonstrated that alkaline phosphatase regulates axonal growth via P2X7R. A study that exposed cultured hippocampal neurons to ATP reported slow axon growth. By contrast, hippocampal neurons cultured with P2X7R antagonists or P2X7R deficiency had faster axon growth and formed more branches [52]. In addition, presynaptic P2X7R regulates the release of neurotransmitters [51]. P2X7R is also involved in the process of neuronal differentiation [48, 53]. However, P2X7R is harmful in pathological states. For example, P2X7R is involved in ATP-induced neuronal death [54]. ATP was found to induce neuronal death in pure culture, and neuronal activity was restored after the use of the P2X7R-specific antagonists A438079 and KN-62, suggesting that P2X7R is involved in ATP-induced neuronal death. In a later study, ATP-induced neuronal death was found to be correlated with high expression levels of P2X7R [55].

### P2X7R in Astrocytes and Oligodendrocytes

In addition to microglia, P2X7R is also expressed by astrocytes [56, 57] and oligodendrocytes [58]. P2X7R mediates multiple physiological functions of astrocytes. For example, the release of glutamate from astrocytes can be involved in signaling between brain cells, and glutamate can also regulate synaptic activity, while P2X7R receptors are involved in the regulation of glutamate release from astrocytes [59]. In addition, P2X7R is also involved in the regulation of ATP release from astrocytes, which participate in intercellular communication through ATP-mediated Ca^2+^ waves [60, 61]. Another important role of P2X7R activation in astrocytes is the upregulation of MCP1/CCL2 expression via the p-38MAPK and ERK1/2 pathways [62, 63]. Functional P2X7 receptors that can mediate cell death in vitro and in vivo are expressed by Schwann cells and oligodendrocytes [58]. More intuitively, P2X7R labeled by functional EGFP was observed in oligodendrocytes of P2X7 BAC transgenic mice [64]. Another study also showed that P2X7R may be involved in oligodendrocyte migration under pathological conditions [65].

## Mechanism of P2X7R Upregulation in Alzheimer’s Disease

Initially, researchers discovered that P2X7R is upregulated in a transgenic mouse model of Alzheimer’s disease [17]. Subsequently, semi-quantitative reverse transcriptase-polymerase chain reaction also revealed enhanced expression of P2X7R in the microglia of AD patients [40]. However, due to the complexity of P2X7 function, the exact mechanism of P2X7 upregulation remains unclear. In general, the regulatory mechanisms of P2X7 expression can be divided into transcriptional and post-translational regulation [66]. The P2X7 promoter contains putative binding sites for several transcription factors, among which specificity protein 1 (Sp1) has been particularly noted as a potential P2X7 transcriptional regulator. Sp1 is a damage-activated transcription factor highly expressed in the brain [67]. Sp1 is involved in the transcriptional regulation of receptors in the central nervous system, and it has been demonstrated that Sp1 is a key component in the transcriptional regulation of P2X7 [68]. It was found that in a mouse model of epilepsy, the transcription factor Sp1 induced P2X7R expression [69]. A recent study reported that progerin 2 deficiency promotes Aβ-induced injury and neuroinflammation by upregulating P2X7R expression via the Sp1 pathway [70]. Inhibition of SP1 may therefore contribute to the upregulation of P2X7R in AD.

## The Relationship Between P2X7R and Aβ

One pathological feature of AD is the extracellular aggregation of Aβ plaques [71, 72]. Aβ peptide is produced by the hydrolytic cleavage of amyloid precursor protein (APP), which is sequentially cleaved by aspartic proteases through amyloidogenic and non-amyloidogenic pathways [73]. In the amyloidogenic pathway, APP is successively hydrolyzed by β-secretase and γ-secretase to produce Aβ, while in the anti-amyloidogenic pathway, APP is cleaved by α-secretase and γ-secretase, ultimately producing p3 and sAPP-alpha, the latter of which has a well-established neuroprotective effect [74, 75].

**Fig. 2:**
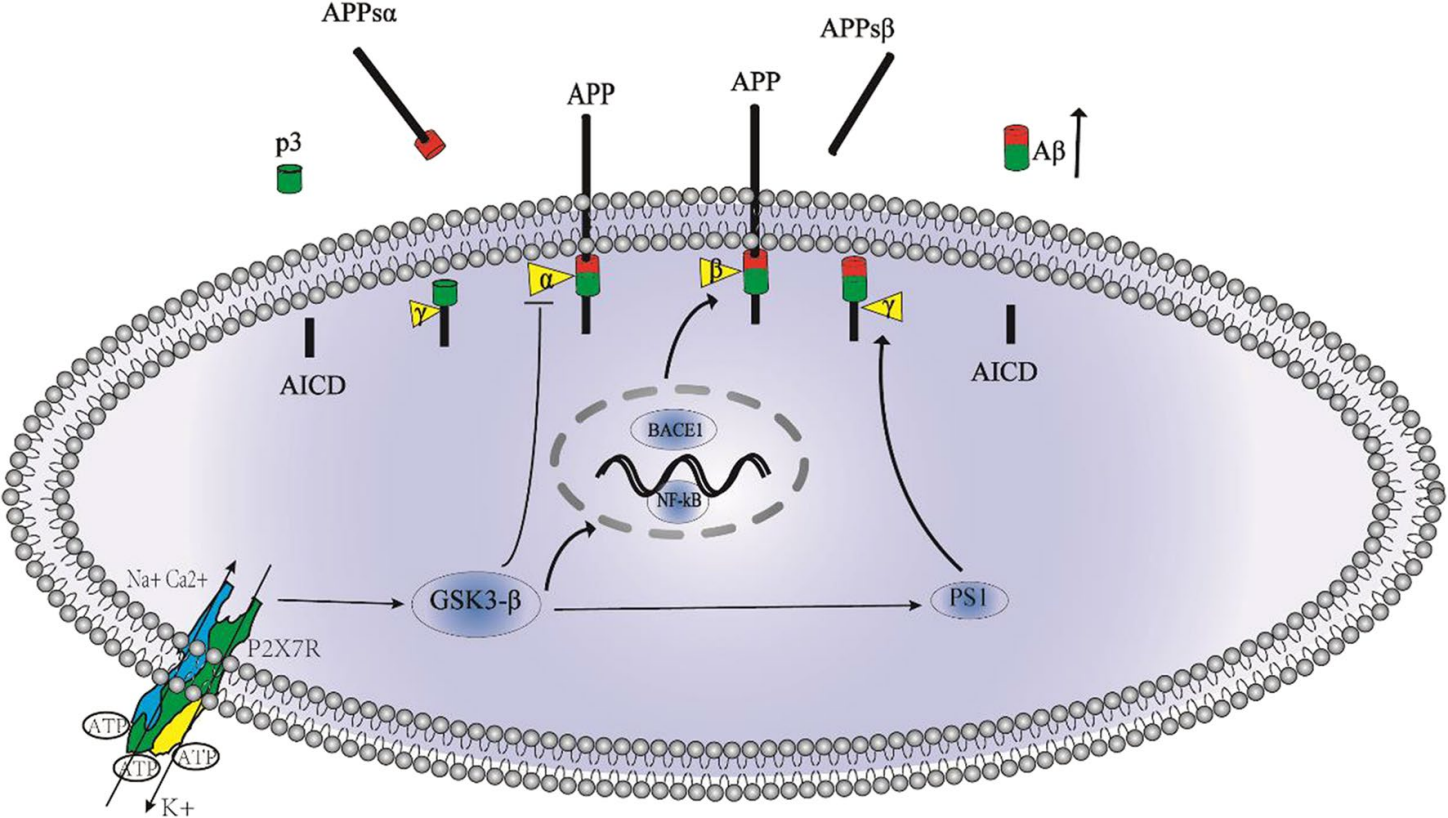
The relationship between P2X7R and Aβ. P2X7R activation promotes Aβ formation by adjusting Glycogen synthase kinase-3β (GSK3-β) activity. (1) GSK3-β induces β-site APP cleaving enzyme 1(BACE1) gene expression through upregulation of nuclear factor kappa-B(NF-kB) signaling; (2) regulates γ-secretase activity by modulating presenilin1((PS1) activity; (3) directly regulates α-secretase activity

Notably, both APP processing modalities can be found in the same cells of the central nervous system [76]. The balance between normal and pathological APP processing is still an active area of research on Aβ accumulation as a characteristic hallmark of AD. The process of Aβ production can be regulated by various signaling pathways. Glycogen synthase kinase-3β (GSK3-β) is considered an important enzyme in AD pathophysiology [77]. In fact, an increase in GSK-3 activity can directly lead to increased accumulation of Aβ [78]. It was shown that inhibition of P2X7R activity in J20 mice reduced Aβ, which was associated with increased α-secretase activity by decreased GSK-3β activity [14]. GSK-3β also interferes with APP cleavage by affecting presenilin1(PS1) activity [79]. Furthermore GSK-3β can mediate β-site APP cleaving enzyme 1(BACE1) expression through nuclear factor kappa-B(NF-kB) [80]. When this balance is upset, the accumulation of Aβ will increase, but P2X7R receptor antagonists can reverse this effect (Fig. 2). It has been shown that the use of P2X7R antagonists reduces GSK-3β activity, which in turn reduces Aβ [14].

In addition, P2X7R activation can also regulate the function of microglia, affecting Aβ. First, P2X7R regulates microglial cell migration, causing microglia to accumulate near senile plaques [37]. In addition, P2X7R also regulates the ability of microglia to phagocytize Aβ [37]. In another study, researchers using BBG, an antagonist of P2X7R, in a mouse model of AD could observe smaller plaques [14].

## The Connection Between P2X7R and Tau

Another characteristic pathological feature of AD is the intracellular aggregation of neurofibrillary tangles (NFT), mainly composed of highly phosphorylated tau protein [81]. Tau is a microtubule-associated protein (MAPT) that polymerizes tubulin into microtubules and participates in sustaining the complex neuronal cell microarchitecture, with roles such as stabilization and microtubule assembly, especially in axons [82].Under pathological conditions, tau proteins dissociate from microtubule-binding proteins and form NFTS [83]. This process is regulated by a variety of enzymes including, but not limited to, C-kinase, A-kinase, cell cycle protein-dependent kinase-5, glycogen synthase kinase-3β [84, 85]. As a phosphoprotein, tau is directly regulated by its phosphorylation state, and NFTs are induced by abnormal Tau phosphorylation, which is modulated by glycogen synthase kinase-3 β (GSK-3β) and cyclin-dependent kinase 5 (CDK5) [86]. GSK-3β is arguably the most widely investigated kinase associated with abnormally high tau phosphorylation [87]. GSK-3β phosphorylates tau mainly by using the PI3K/ AKT/GSK-3β pathway [87]. Studies have shown that P2X7R promotes neuronal tau phosphorylation via GSK3 kinase [52]. What’s more, the tau-related pathology of AD is also affected by inflammation, and tau propagation is suppressed by the depletion of microglia [88]. P2X7R plays an important role in this process. Elevated P2X7R expression was found in the brains of transgenic mice and patients with tauopathies. Moreover, hippocampus-dependent spatial memory and long-term synaptic plasticity were improved by P2X7R deletion in a mouse model of tauopathy [15, 89]. In a tauopathy mouse model, researchers found that oral administration of GSK1482160, a P2X7R-specific antagonist, significantly improved cognitive performance in rats [90]. Similarly, blockade of IL-1β signaling leads to reduced tau pathology [91]. Conversely, tau pathology was exacerbated by persistent interleukin-1β overexpression [92]. Microglial activation has been shown to precede tau pathology in the P301S mouse model [93]. Studies have demonstrated that microglial activation significantly accelerated tau pathology and behavioral abnormalities in model mice [94]. Reactive microglia drive tau pathology and it help spread abnormal tau in the brain [95]. Pharmacological blockade of P2X7R decreased the accumulation of misfolded tau aggregates and restored cognitive function in P301S mice, likely by suppressing exosome secretion [90]. Studies have shown that the use of P2X7R antagonists reduces cell death by altering the balance of tau phosphorylation inside and outside the cells [96]. The same study also found that P2X7R inhibition affected extracellular tau phosphorylation by reducing tissue-nonspecific alkaline phosphatase (TNAP) expression.

## P2X7R and Neuroinflammation in AD

Neuroinflammation is considered to be the third core pathology of AD, and inflammation is involved in the onset as well as the progression of the disease. Moreover, the neuroinflammation hypothesis also links the two other hypotheses of AD pathogenesis [97]. As resident immune cells of the CNS, microglia are key regulators of neuroinflammation [98]. As such, microglia are involved in the maintenance of the inflammatory and immune response in the entire brain [99]. In response to different environmental factors and stimuli, microglia can usually be activated into two polarized states, called the M1 phenotype and M2 phenotype [100, 101]. M1 microglia are thought to enhance the inflammatory response, while M2 microglia exert neuroprotective effects and promote tissue repair by inhibiting neuroinflammation. M1 microglia can secrete a variety of pro-inflammatory cytokines and chemokines, such as IL-1β, IL-6, IL-18, and TNF-α [102, 103]. IL-1β plays an important role in inflammation. The activation of the NLRP3 inflammasome in microglia is the basis for the maturation and release of IL-1, IL-6,IL-18, and TNF-α [104], and P2X7R is involved in regulating NLRP3. Therefore, P2X7R is essential for the release of pro-IL-1β from microglia [16, 43]. The production of IL-1β is a two-step process. First, a large amount of Pro-IL-1βis synthesized in the cytoplasm and is then proteolytically processed into IL-1B before it is finally released. Even LPS-mediated synthesis of large amounts of pro-IL-1β requires the activation of the P2X7 receptor to release mature IL-1β [105]. This also illustrates the importance of P2X7R in the maturation process of pro-inflammatory cytokines. The release of IL-1 β by microglia is mainly dependent on NLRP3 activation. P2X7R activation leads to K^+^ efflux, after which reduced intracellular K^+^ initiates NLRP3 inflammasome assembly and activation [106, 107]. The NLRP3 inflammasome consists of the adapter protein ASC (apoptosis-associated speck-like protein containing a CARD) and the sensor protein NLRP3. ASC can recruit and activate pro-caspase-1, which can be cleaved by the induced complex to produce active caspase-1, which in turn cleaves pro-IL-1β and IL-18, eventually leading to the release of IL-1 [108]. A defective NLRP3 inflammasome was found to result in reduced Aβ deposition in the APP/PS1 model of Alzheimer’s disease [109]. All these studies suggest that the NLRP3/caspase-1 axis may be a target for the treatment of Alzheimer’s disease.

## P2X7R and Mitochondrial Dysfunction in Alzheimer’s Disease

Alterations in energy metabolism occur in their brains during the early stages of AD, and the researchers used fluorodeoxyglucose positron emission tomography to monitor hypometabolism of glucose in the brain [110]. This implies that mitochondrial dysfunction is extremely important in the development of the disease. In amyotrophic lateral sclerosis, P2X7R was shown to be involved in autophagy in microglia [111]. Not only that, the P2X7R signaling pathway mediates impairment of lysosomal function [112]. The adenosine monophosphate (AMP)-activated protein kinase (AMPK) pathway is closely related to mitochondrial division [113]. Researchers find that P2X7R activation can induce mitochondrial fission and affect mitochondrial autophagy through an AMPK-dependent pathway [114]. Mitochondrial autophagy maintains a balance between mitochondrial production and mitochondrial death. However, in AD, mitochondrial autophagy is impaired, which in turn affects energy metabolism [115, 116]. Impaired mitochondrial autophagy impairs mitochondrial autophagy by increasing oxidative damage and cellular energy deficits cognitive deficits, which in turn impair mitochondrial autophagy [117].

Oxidative stress is a condition caused by the increased production of reactive oxygen species (ROS) that is greater than the capacity of cellular antioxidant mechanisms. ROS are normally produced during physiological processes and have both beneficial and harmful effects in biological systems [118]. In AD, the apparent oxidative imbalance and increase of ROS have been widely noted [119]. A study comparing the levels of isoprostane 8,12-iso-iPF(2alpha)-VI in cerebrospinal fluid, plasma, and urine of cognitively normal elderly and probands with mild cognitive impairment (MCI) found significantly elevated levels of these substances in MCI. The isoprostane 8,12-iso-iPF(2alpha)-VI is a specific marker of in vivo lipid peroxidation. This also suggests a significant oxidative imbalance in the early stages of AD when there is no significant accumulation of senile plaques and NFTs [120, 121]. This suggests that the onset of oxidative stress precedes the development of AD-related pathology and may contribute to its development. In addition, high levels of ROS are often detected in the brains of patients with different types of neurodegenerative diseases [122]. In addition, a variety of blood markers of oxidative stress, such as protein carbonyl and 3-nitrotyrosine, are frequently detected in AD patients or corresponding animal models [123, 124]. Although ROS are mainly produced by mitochondria, which have strong innate oxidation resistance, excessive ROS accumulation can also cause mitochondrial dysfunction, which is also one of the prominent pathological features of AD [125].

Notably, a recent study demonstrated the mitochondrial localization of the P2X7R ionotropic purinergic receptor [126]. In addition, the basal respiration rate, ATP-coupled respiration, maximal uncoupled respiration, resting mitochondrial potential, and mitochondrial matrix Ca^2+^ levels were affected when there was no P2X7R in the mitochondria. This may indicate that P2X7R is also an improtant regulator of mitochondrial energy metabolism. The researchers also found reduced proteasome activity in the brains of AD patients as well [127]. The latest study found that sustained activation of P2X7R can induce ubiquitin–proteasome system dysfunction and ultimately lead to neuronal death [128]. This implies that P2X7R is involved in multiple metabolic mechanisms in the brains of AD patients.

In addition, it was shown that P2X7R expression in the APPswe/PS1dE9 mouse model of Alzheimer’s disease may mediate neuronal injury through the generation of ROS [39]. It was also confirmed in another study that Aβ can induce mitochondrial toxicity, but this process requires the involvement of P2X7R in microglia [129]. Furthermore, activation of P2X7R in the microglial cell membrane by ATP may induce the generation of hydrogen peroxide [130]. Recent research has demonstrated that P2X7R activation following stimulation with BzATP or ATP can induce ROS generation in microglia and macrophages. And the researchers found that the use of P2X7R inhibitors reduced the production of ROS [17, 131, 132]. Mitochondrial dysfunction along with oxidative stress appears to be an important early event in disease development.

## P2X7R and Synaptic Dysfunction in AD

AD is also characterized by synaptic loss and dysfunction. Synaptic loss and dysfunction in the brain can be detected by researchers early in the progression of AD disease and is strongly associated with cognitive decline in AD patients [133]. By directly observing neurons in AD transgenic mice, the researchers found a significant reduction in spine density [134]. Reduced synaptic density and fewer synaptic connections per neuron can also be observed in the AD brain [135]. In addition, synaptic dysfunction is one of the earliest hallmarks of AD. In the early stages of AD, the decrease in synaptic proteins precedes all other neurodegenerative markers in the cerebrospinal fluid [136].

P2X7R is likely to be involved in synaptic changes in AD progression. In a mouse model of AD, researchers show that synaptic dysfunction may be linked to ROS production by P2X7R [39]. Meanwhile, the inhibition of P2X7R favors the reduction of aβ and Tau. aβ and Tau play a role in synaptic damage [137]. P2X7R is also involved in regulating synaptic neurotransmission. P2X7R can excite synapses by mediating the release of glutamate from astrocytes [59]. Further studies, the researchers found that the use of BBG, an antagonist of P2X7R, improved the development of dendritic spines in hippocampal neurons of AD model mice [138].

## P2X7R as a Potential Target in AD

**Fig. 3:**
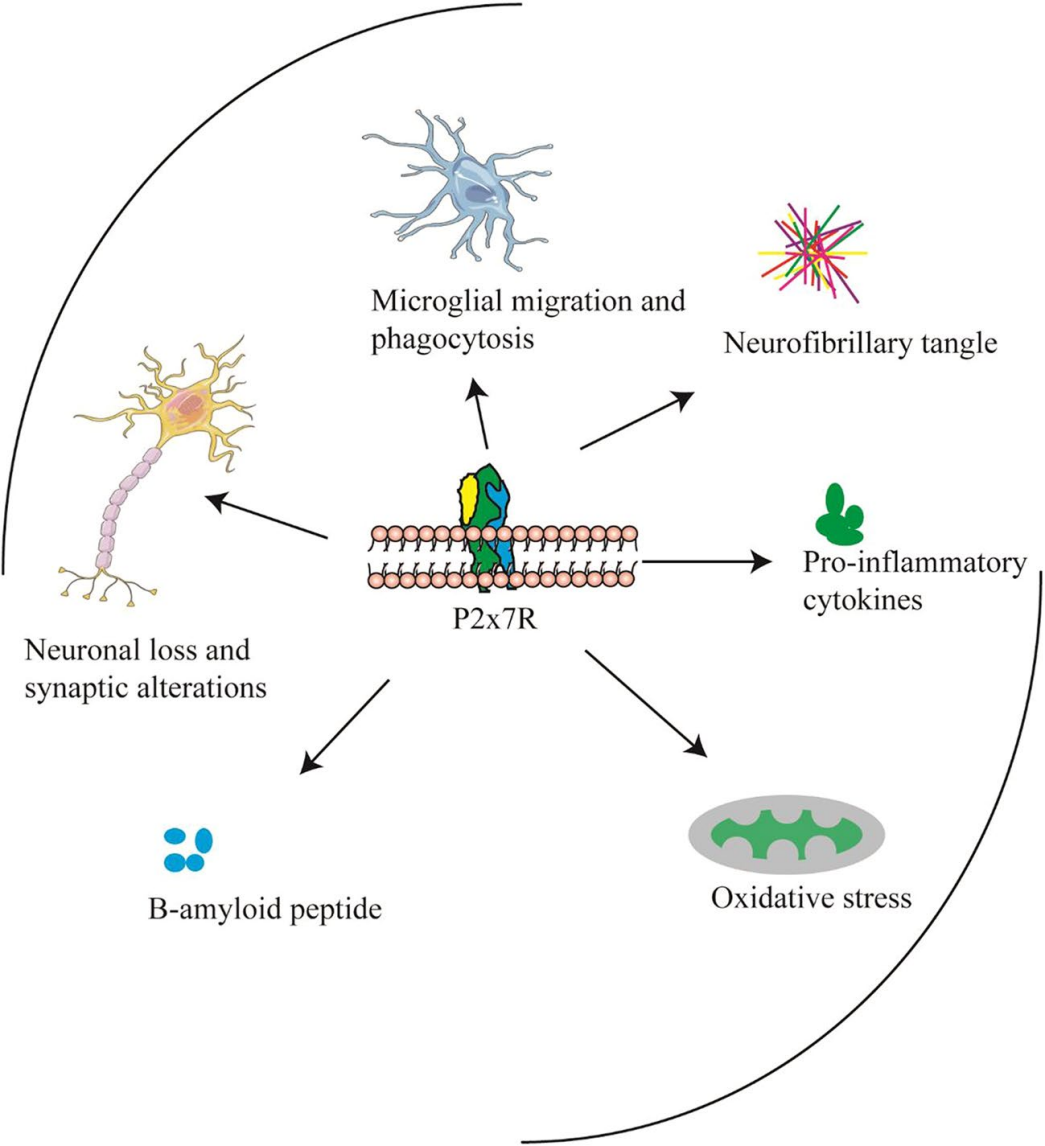
This figure suggests that P2X7R is involved in different physiopathological processes in Alzheimer’s disease. As the figure demonstrates, P2X7R regulates the processing of amyloid APP, promotes Tau phosphorylation, and is also involved in synaptic changes, ROS, microglia activation, and promotes inflammatory factor release which are all processes that contribute to AD progression

AD is now increasingly recognized as a multifactorial neurodegenerative disease with different pathological processes as possible contributors, including amyloid deposition, tau protein phosphorylation, oxidative stress, or chronic neuroinflammation. Notably, P2X7R is involved in all these processes (Fig. 3) and consistently appears to play an important role in the development of Alzheimer’s disease. The fact that P2X7R is antagonistic only when activated at high ATP concentrations is a significant advantage for a drug target [19]. In this way, pharmacological antagonism of P2X7R will not affect the function of P2X7R in normal physiological states. It was reported that in vivo P2X7R inhibition reduced amyloid plaques, providing the first evidence of the potential of P2X7R antagonists as therapeutic agents for Alzheimer’s disease [14]. Similarly, P2X7R antagonist treatment was also found to prevent the development of amyloid plaques in a mouse model of AD. Moreover, cognitive decline was rescued by P2X7R knockout in the APP/PS1 mouse model of AD [139].

The potential of P2X7R as a therapeutic target is supported by reports of improved symptoms and neuropathology in animal models of AD through pharmacological blockade or gene deletion [138–140]. For example, one study showed that the P2X7R antagonist BBG rescued spatial memory, learning, and cognitive deficits in a mouse model of AD [138]. BBG is a derivative of Brilliant Blue FCF, which has been proven safe in healthy animals and is approved for use as a food additive in the USA under various brand names, such as FD&C Blue No. 1 or Acid Blue 9 [141]. Notably, BBG also has the advantage of high blood–brain barrier permeability [142]. In the brain, BBG not only reduces the level of purinoceptor expression but also attenuates gliosis [143]. Moreover, BZ-ATP treatment increased IL-1β secretion in human microglia that had been preactivated with Aβ(1–42), while pretreatment with P2X7 receptor antagonists had the opposite effects [144]. In addition to treatment with P2X7R antagonists, inhibition of the P2X7R pathway may be a new therapeutic approach for the treatment of AD. Thus, P2X7R has great therapeutic potential.

## Advances in Drug Research

The potential of P2X7R as a drug target has long been noted in other diseases, such as neuropsychiatric disorders, and cancer [145, 146]. Surprisingly, P2X7R antagonists also have neuroprotective effects in central nervous system disorders. It has shown great potential in both multiple sclerosis and ALS [147, 148]. BzATP is a P2X7R agonist. It elicits pore formation, IL-1β release, and calcium influx in rats, human receptors, and mice. Researchers have investigated a variety of P2X7R antagonists. The species-dependent differences in receptor sensitivity have been well summarized for the various P2X7R antagonists [149]. Although most P2X7R antagonists do not cross the blood–brain barrier and are unable to act in vivo, recent studies have made significant progress in identifying brain-permeable P2X7R antagonists. GlaxoSmithKline has developed the P2X7 receptor antagonist GSK1482160 with good CNS penetration [150]. The amide GSK1370319A also showed good brain penetration [151]. It inhibits inflammasome-induced cell death and neurodegeneration [152]. JNJ-54175446 and JNJ-55308942 are two new brain-penetrant P2X7R antagonists [153, 154]. More brain penetrant P2X7R antagonists are listed in Table 1. For more details see Table 1.

**Table 1:** Features of various brain-permeable P2X7R antagonists

Class/Compound	Features	Ref
JNJ-54175446	Progressed into preclinical development	[154]
JNJ-55308942	Retained rodent activity	[153]
JNJ-47965567 and JNJ-42253432	Shows activity on rodent and human P2X7	[155, 156]
JNJ-54175446	High affinity and potency P2X7 antagonist	[157]
GSK1482160	Good CNS permeability	[150]
BBG	The selective antagonist for P2X7R	[142]

## P2X7R as a New Diagnostic Tool for AD

PET imaging is a recognized technique commonly used to diagnose brain diseases including Alzheimer’s disease [158]. PET studies of Alzheimer’s disease have the advantage of identifying different subtypes of the disease through neuropathology and sometimes genetic causation, which may have implications for guiding treatment. PET imaging of Alzheimer’s disease has so far mainly used the radiotracer [18F]FDG to image glucose metabolism [159]. However several radiotracers have been developed for the detection of other molecules, interestingly including several P2X7R radiotracers [146]0.11C-JNJ-54173717 was developed as a high-affinity P2X7R antagonist. In animal rat models, it has demonstrated its advantage as a PET radioligand for visualizing the expression and distribution of P2X7R in vivo. And it can also be used in monkey brain to selectively display P2X7RX expression and distribution [160]. 18F-JNJ-64413739 is also considered a suitable PET ligand for quantifying P2X7R expression in the human brain, which can be used for P2X7R expression in health and Alzheimer’s disease to provide ideas for therapy [161]. However, whether P2X7R-PET has the potential to stratify Alzheimer’s disease such as disease severity needs to be analysed in a larger cohort of patients, but P2X7R-based PET imaging may be a promising tool.

P2X7R is not only expressed in brain tissue but also in the peripheral immune system, where it can be found in macrophages and T cells [162, 163]. A recent study has shown that the expression of P2X7R is elevated in the blood of patients with Alzheimer’s disease, so it can be assumed that the plasma level of P2X7R is a biomarker that can differentiate between patients with Alzheimer’s disease and non-Alzheimer’s disease [164]. Although we should not use a single biomarker as a diagnostic tool, elevated plasma levels of P2X7R in patients with Alzheimer’s disease suggest that combined plasma levels of P2X7R are promising as a diagnostic tool.

## Conclusions and Prospects

We summarized the roles of P2X7R in the central nervous system and its significance in the pathogenesis of AD, after which we discussed the various effects of P2X7R activation. As described in this review, the success of P2X7R antagonists in preclinical models indicates that P2X7R should be a focus of future research on targeted therapies for AD. A detailed understanding of the roles of P2X7R is essential for the discovery of new therapeutic approaches for the treatment of neurological disorders. A great potential advantage may lie in the absence of P2X7R activation or low P2X7R expression in healthy tissues, which may limit the side effects of drug treatment.

Although many P2X7R antagonists have now been developed, many challenges remain. Highly selective and effective P2X7R agonists and antagonists that can penetrate the CNS need to be further explored. Importantly, the clinical use of these drugs will first require extensive further study of their safety. In addition to treatment with P2X7R antagonists, inhibition of the P2X7R pathway may also be a new therapeutic approach for the treatment of AD. With further exploration of AD pathogenesis and further drug development, P2X7R-targeted therapies are likely to become a promising new treatment modality in the future.

## Data Availability

Not applicable for that section.
